# Extensive purifying selection acting on synonymous sites in HIV-1 Group M sequences

**DOI:** 10.1186/1743-422X-5-160

**Published:** 2008-12-23

**Authors:** Nobubelo K Ngandu, Konrad Scheffler, Penny Moore, Zenda Woodman, Darren Martin, Cathal Seoighe

**Affiliations:** 1 National Bioinformatics Network Node, Institute of Infectious Diseases and Molecular Medicine, Faculty of Health Sciences, University of Cape Town, Anzio Road, Observatory, 7925, South Africa; 2 Computer Science Division, Dept of Mathematical Sciences, University of Stellenbosch, Private Bag X1, 7602 Matieland, Stellenbosch, South Africa; 3 National Institute for Communicable Diseases, Private Bag X4, Sandringham, Johannesburg, 2131, South Africa; 4 HIV Diversity and Pathogenesis Group, Institute of Infectious Diseases and Molecular Medicine, Faculty of Health Sciences, University of Cape Town, Anzio Road, Observatory, 7925, Cape Town, South Africa

## Abstract

**Background:**

Positive selection pressure acting on protein-coding sequences is usually inferred when the rate of nonsynonymous substitution is greater than the synonymous rate. However, purifying selection acting directly on the nucleotide sequence can lower the synonymous substitution rate. This could result in false inference of positive selection because when synonymous changes at some sites are under purifying selection, the average synonymous rate is an underestimate of the neutral rate of evolution. Even though HIV-1 coding sequences contain a number of regions that function at the nucleotide level, and are thus likely to be affected by purifying selection, studies of positive selection assume that synonymous substitutions can be used to estimate the neutral rate of evolution.

**Results:**

We modelled site-to-site variation in the synonymous substitution rate across coding regions of the HIV-1 genome. Synonymous substitution rates were found to vary significantly within and between genes. Surprisingly, regions of the genome that encode proteins in more than one frame had significantly higher synonymous substitution rates than regions coding in a single frame. We found evidence of strong purifying selection pressure affecting synonymous mutations in fourteen regions with known functions. These included an exonic splicing enhancer, the rev-responsive element, the poly-purine tract and a transcription factor binding site. A further five highly conserved regions were located within known functional domains. We also found four conserved regions located in *env *and *vpu *which have not been characterized previously.

**Conclusion:**

We provide the coordinates of genomic regions with markedly lower synonymous substitution rates, which are putatively under the influence of strong purifying selection pressure at the nucleotide level as well as regions encoding proteins in more than one frame. These regions should be excluded from studies of positive selection acting on HIV-1 coding regions.

## Background

Several statistical models of codon evolution have been developed and applied to protein-coding sequences from viral and other pathogens [1-4]. The primary application of these models has been the detection of evidence of diversifying selection acting on protein coding DNA sequences. Within maximum likelihood or Bayesian frameworks these models can be used to identify specific sites at which adaptive mutations have occurred. In the context of virus infections this information can be especially useful for identifying immune escape and drug resistance mutations [3,5,6].

Positive selection is frequently inferred by comparing the rate of non-synonymous substitutions per non-synonymous site (dN) to the rate of synonymous substitutions per synonymous site (dS). The ratio of these two rates is often represented by the symbol ω. Under the assumption that synonymous substitutions are neutral and that the synonymous substitution rate therefore approximates the neutral rate of evolution, diversifying selection can be inferred when ω is greater than one. Several methods exist to determine whether there is evidence that ω is greater than one at a subset of sites in a protein-coding gene (i.e. the gene is evolving under diversifying selection) and to identify the sites within the gene at which diversifying selection occurs [3,4,7-9].

In many of the situations in which this strategy is applied, the assumption that synonymous substitutions are fixed at a constant rate and provide a good estimate of the neutral rate of evolution, may not hold. Kosakovsky Pond & Muse reported that coding sequences from a wide range of taxa, including HIV-1, show strong evidence of variation in the rate of synonymous substitution across coding regions [10]. There are two possible causes of synonymous rate variation. If synonymous substitutions are indeed neutral, variation in the mutation rate can cause the synonymous substitution rate to vary. In such a case, it is possible to include a varying synonymous substitution rate in the codon models of evolution and inference of positive selection from comparison of the local synonymous and nonsynonymous substitution rates remains feasible. However, if the variation in synonymous substitution rate is caused by selection acting to preserve functions that are encoded at the nucleotide level, even a comparison of local nonsynonymous and synonymous substitution rates cannot be used to infer positive selection because the synonymous substitution rate is no longer a valid proxy for the neutral rate of evolution and the standard approach of inferring the action of diversifying selection when ω > 1 is not valid.

Failure to model variation in synonymous substitution rate will result in an overall underestimate of the neutral rate of evolution. This undermines the validity of the inference of selection, because nonsynonymous substitution rates are compared against a rate which is no longer a good estimate of the neutral rate, and this is likely to result in inference of diversifying selection at a proportion of the sites that are actually evolving neutrally. Indeed, as the number of taxa increases, we expect an ever greater proportion of the neutral sites to be classified as diversifying selection sites in this scenario. Alternatively, if the synonymous substitution rate variation is modeled and selection inferred when dN is greater than the local dS rate then we expect a very high probability of false inference of selection at codons where the synonymous positions happen to be functionally important and conserved, and the nonsynonymous positions are neutral or experience less purifying selection. Thus, in general, in a codon-based method, analysis of selection is unreliable when there is purifying selection acting to preserve functions at the nucleotide level. An example where an elevated ω was attributable to purifying selection acting on synonymous sites was reported by Hurst & Pal [11].

Several examples of sequence motifs within protein-coding sequences that are expected to be under purifying selection at the nucleotide level are known in HIV-1, many of which are involved in regulating gene expression. Examples include the 3' long terminal repeat (LTR) region, part of which also encodes the Nef protein [12,13]. In addition to conserved RNA secondary structures, the LTR contains several regulatory elements, some of which directly interact with cellular transcription factors (e.g. the Ets protein family) [14,15]. The viral sequence also has an intragenic nuclease hypersensitive region involved in regulating gene expression (also referred to as HS7) in the *pol *gene [16-18] and the rev-responsive element (RRE) in the *env *gene which interacts with the Rev protein to transport unspliced or partially spliced RNA from the nucleus to the cytoplasm of the infected cell [17,19-21]. Some functionally important regions of the RRE have previously been found to be conserved at the nucleotide sequence level, presumably the result of purifying selection pressure to preserve this function [22]. The inhibitory sequence elements (INS) in gag and the cis-repressive sequence (CRS) in *pol *are examples of negative regulators of transcription [23-26]. If these functional sites are important for viral viability, then we expect them to be preserved by purifying selection.

While it represents a significant challenge for studies of selection acting on the HIV-1 amino acid sequence, the variability in the synonymous substitution rate may also provide useful information about previously unknown sequence motifs within the coding fraction of the HIV-1 genome that function at the nucleotide level. Although some variability can be explained by a variable mutation rate, the identification of regions of very high conservation that cannot be explained by selection acting on the amino acid sequence or by known motifs that function at the nucleotide level has the potential to highlight novel functions encoded in the HIV-1 genome.

Here we use an existing model of codon sequence evolution [10] to provide the first complete overview of site-to-site variation in synonymous substitution rate across the whole HIV-1 genome and identify selection pressures likely to be driving this variation. This model allows dN and dS to vary independently across sites, ensuring that the estimated dS values reflect selection pressure acting upon the nucleotide sequence and not at the amino acid level. It is worthwhile to distinguish between selective pressure acting at the nucleotide level, affecting both synonymous and nonsynonymous changes, and at the amino acid level, affecting nonsynonymous changes only. Unfortunately, quantifying the relative contributions of nucleotide and amino acid level effects on nonsynonymous changes is highly sensitive to model assumptions. We therefore restricted the analysis to synonymous changes and do not attempt to quantify the nucleotide-level selective pressure on nonsynonymous changes. We took into account recombination breakpoints in order to avoid biased estimates that can result from fitting phylogenetic models that do not take recombination into account [27,28].

We report patterns of sequence conservation around nucleotide sequence motifs with known functions and identify additional conserved nucleotide elements that do not fall within any currently characterized functional motifs. Finally, we report the locations of all HIV-1 genome regions where we infer that purifying selection acting directly on the nucleotide sequence is likely to cause a substantial reduction in the synonymous substitution rate. These are provided with respect to the HXB2 reference strain, to enable other researchers to mask these regions from their analyses of positive selection acting on HIV-1 genes.

## Methods

### Sequence data

Nucleotide sequence alignments consisting of HIV-1 Group M subtype reference sequences were downloaded from the Los Alamos database  for each gene of the HIV-1 genome [29]. Each alignment had at least one sequence (total ranging from 32 to 37) from each of the 11 non-recombinant HIV-1 group M subtypes A1, A2, B, C, D, F1, F2, G, H, J and K [Genbank: AB253421, AB253429, AF004885, AF005494, AF005496, AF061641, AF061642, AF067155, AF069670, AF075703, AF077336, AF082394, AF082395, AF084936, AF190127, AF190128, AF286237, AF286238, AF377956, AF484509, AJ249235, AJ249236, AJ249237, AJ249238, AJ249239, AY173951, AY253311, AY331295, AY371157, AY371158, AY423387, AY612637, AY772699, DQ676872, DQ853463, K03454, K03455, U46016, U51190, U52953, U88824, U88826]. All regions encoding amino acids in more than one frame were identified and regions judged by eye to be unreliably aligned, i.e., positions 6544–6595, 6700–6715, 7318–7375 of the *env *gene region, were excluded from the analysis. We used the HIV-1 genome map and sequence annotations available from the Los Alamos database to identify the regions of the genome that encode proteins in a single reading frame (see Table 1) [29].

**Table 1:** Summary of HIV-1 reference sequence data used

***Gene***	***Non-overlapping region used ***	***Position on genome***	***Number of sequences***
*env*	88 – 2154	6313 – 8379	37
*gag*	1 – 1295	790 – 2084	37
*nef*	1 – 621	8797 – 9417	32
*pol*	211 – 2955	2296 – 5040	37
*tat*	22 – 138	5851 – 5967	37
*vif*	58 – 519	5098 – 5559	37
*vpr*	61 – 273	5620 – 5832	37
*vpu*	1 – 162	6061 – 6222	37
*Rev*	total overlap		
*genome*	Includes overlapping sites	790 – 9417	36

The HXB2 genome numbering is used.

We identified recombination breakpoints in each alignment using the GARD (Genetic Algorithm for Recombination Detection) algorithm implemented in the HyPhy (Hypothesis testing using Phylogenies) package [30,31]. Evidence of recombination was detected in all genes except *tat*, *vpr *and *vpu*. GARD outputs both an alignment showing the positions of recombination breakpoints and separate tree topologies for each of the sequence alignment segments bounded by these breakpoints.

### Synonymous substitution rate estimation

Synonymous substitution rates were estimated using a version of the MG94 codon substitution model [1,10]. We used the Dual Model, which allows dS to vary independently of dN and used three discrete categories for each rate. We ran the selected models using a HyPhy batch script for analysis of selection acting on recombining sequences which we developed previously [32]. This method uses separate tree topologies for each partition of the sequence alignment while keeping the rest of the model parameters fixed across all partitions. We used sliding window plots of mean dS values, calculated over three adjacent codons, to identify regions with low synonymous substitution rates.

We also analyzed an alignment of HIV-1 subtype C *gag *sequences [Genbank: DQ792982-DQ793045] described previously [33] to assess the impact of conservation acting on synonymous substitutions on inference of positive selection. We inferred positive selection using model M2a of Yang and colleagues [34], taking recombination into account [32].

### Simulations

We used HyPhy to generate simulated data under a neutral model with trees generated from the original alignments (or the tree from the largest un-recombined region for alignments where recombination was detected). The same sequence alignments used as input in the initial analysis were used and one hundred simulated datasets were generated for each alignment. Each simulated dataset was then analyzed using the Dual Model as described above. For each gene the minimum value of mean dS across all sliding windows of three adjacent codons, in all of the one hundred simulated datasets, was used as a conservative threshold to identify windows of reduced dS in the observed data. This stringent threshold and a less stringent one that included 95% of the values inferred from the simulated data are shown in the sliding window plots.

### Functional analysis of a novel nucleotide sequence motif in *env*

JC53-bl and 293T cells were obtained from Dr George Shaw (University of Alabama, Birmingham, AL) and cultured as described previously [35]. M7-Luc cells (5.25.EGFP.Luc.M7) were kindly provided by Dr Nathaniel Landau through the AIDS Research and Reference Reagent Program, Division of AIDS, NIAID. M7-Luc cells were cultured in RPMI-1640 medium containing 2 mM L-glutamine, 25 mM HEPES, 10% heat-inactivated fetal bovine serum (FBS) and 50 ug/ml gentamicin (Sigma), supplemented with 10 μg/ml DEAE-Dextran for infectivity assays. An infectious molecular clone, p81, was obtained through the AIDS Research and Reference Reagent Program, Division of AIDS, NIAID, NIH: p81A-4 (Cat#11440) from Dr. Bruce Chesebro. Mutations were introduced into p81 using the Stratagene QuickChange XL kit. Infectious viral particles were produced by transfection of 293T cells using Fugene reagent (Roche BioSciences), and transfection output was assessed by determination of TCID_50 _in JC53-bl cells as described previously [35]. Equivalent numbers of TCID_50 _were used to infect M7-luc cells seeded into 96 well plates at a density of 7.5 × 10^5 ^cells/ml followed by a washout step12 hours post-infection. Replication was monitored by measurement of p24 production using the Vironostica HIV-1 antigen Microelisa system (Biomerieux) over 5 days.

## Results

**Figure 1:**
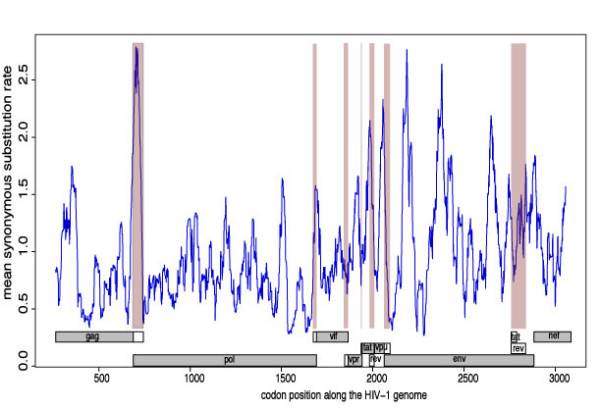
**HIV-1 genome-wide plot of mean nonsynonymous substitution rates**. A 30 codon sliding window was used. Regions coding for proteins in more than one frame are shaded in pink. The frames that were used in each region are shown in grey rectangles, with frame 1 at the top and frame 3 at the bottom.

**Figure 2:**
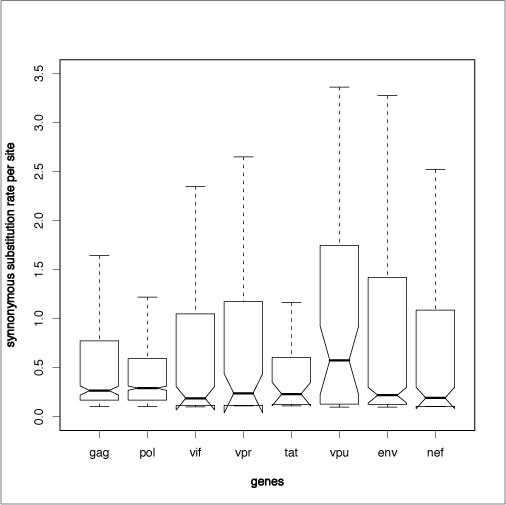
**Box-and-whisker plot showing variation of dS values per gene**. The values are from non-overlapping regions of HIV-1 genes.

Consistent with previous reports [5,10], we found evidence of variation in synonymous substitution rates within and across HIV-1 genes (Figure 1). For all genes the Dual Model [10], which allows independent variation of dS and dN had a much better fit to the data than a model with constant dN and dS (referred to as the Constant model in Table 2) or than a model in which only dN varied across sites (the Nonsynonymous model in Table 2). The variance of dS gives an indication of the extent of site-to-site synonymous rate heterogeneity within the different genomic regions (Table 3). There was significant variation between genes (p-value = 2 × 10^-7^, from Levene's test) with the least site-to-site variation in dS observed in *vpu *and the most in *vpr *followed by *nef *and *env *(Figure 2).

**Table 2:** AIC model selection index to show how different models fit to the data.

**Gene regions**	**Akaike Information Criterion index (AIC) per Model**		
	**Dual**	**Nonsynonymous**	**Constant**

Gag	23862.95	23963.83	25889.10
Pol	41504.20	41668.99	45642.96
Vif	9794.65	9843.41	10502.37
Vpr	9581.57	9692.29	10869.78
Tat	2834.49	2878.99	3110.23
Vpu	11701.57	11832.41	12938.54
Env	45201.62	45422.72	48658.16
Nef	13984.66	14061.46	14986.75

The Dual Model which allows dS to vary across sites has the lowest AIC (best fit to the data) for all 8 genes.

**Table 3:** Regions of the HIV-1 sequence which should be considered for exclusion in positive selection analysis studies.

**Gene**	**dS Variance**	**Overlapping regions**	**Highly conserved (most stringent cutoff)**	**Other sites conserved at 0.05 significance**
*gag*	2.1	2086 – 2295 (gag/pol)	793 – 807	821–823
			898 – 903	1036–1038
			985 – 996	1810–1812
			1309 – 1314	1831–1833
				1969 – 1974
				2002–2004
				2023 – 2025
*pol*	1.8	5041 – 5097 (pol/vif)	4092 – 4094	2490–2495
			4764 – 4790	2850 – 2858
			4864 – 4866	3252 – 3257
			4926 – 4937	3304 – 3312
				3867 – 3881
				3966 – 3971
*vif*	2.7	5560 – 5619 (vif/vpr)	-	-
*vpr*	3.9	5833 – 5850 (vpr/tat)	5769 – 5777	-
			5794 – 5805	
*tat*	2.9	5968 – 6060 (tat/rev)	5855 – 5863	-
			5957 – 5968	
*vpu*	1.4	6223 – 6312 (vpu/env)	6101 – 6106	-
			6143 – 6151	
			6167 – 6178	
*env*	3. 2	8380 – 8796 (env/rev)	7656 – 7667	7077 – 7082
			7834 – 7842	7125 – 7130
			8349 – 8354	7629 – 7634
			8376 – 8378	
*nef*	3.6		9067 – 9086	8869 – 8874
		-	9087 – 9093 (nef/LTR)	8887 – 8892 9121 – 9126
			9183 – 9192 (nef/LTR)	9235 – 9237
			9391 – 9399 (nef/LTR)	

Co-ordinates are adapted from the HXB2 numbering system. Dashes indicate that there are no sites within that category for a particular gene.

**Figure 3:**
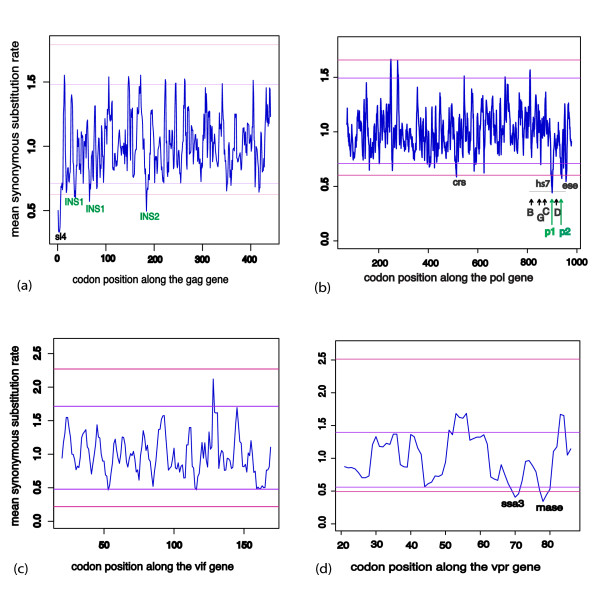
**Mean (blue) synonymous substitution rates observed across *gag, pol*, *vif and vpr *genes**. Mean dS was calculated over sliding windows of three codons. Horizontal lines mark the most stringent (red) and less stringent (purple) significance thresholds. (a) dS across the *gag *gene. 'sl4'; the fourth stem loop of the encapsidation signal, 'INS1'; a motif within the first inhibitory sequence region, 'INS2'; a motif within the second inhibitory sequence region. (b) dS across the *pol *gene. 'crs'; start of the cis-repressive sequence, horizontal dotted line is the nuclease hypersensitive region and sites 'B', 'G', 'C' and 'D' are confirmed transcription factor binding sites known as site-B, GC-box, site-C and site-D respectively. 'p1'and 'p2'; conserved sites within nuclease hypersensitive region. "ese"; exonic splicing enhancer. (c) dS across the *vif *gene. (d) dS across the *vpr *gene. 'ssa3'; 3' splice acceptor site A3, 'rnase'; RNae-V1 cleavage site.

**Figure 4:**
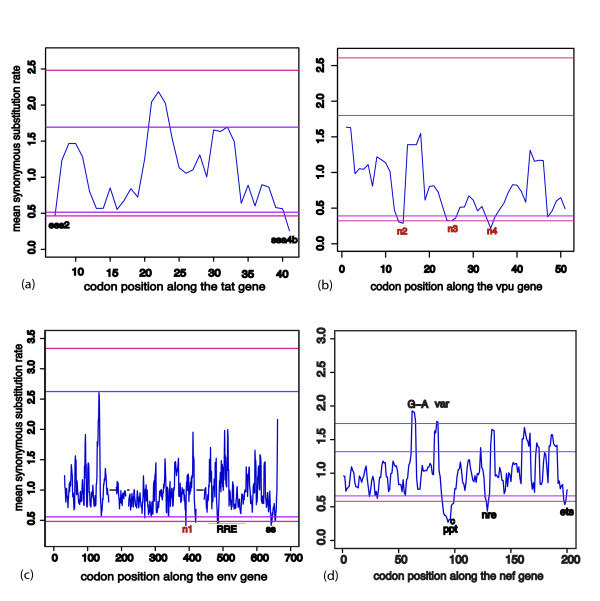
**Mean (blue) synonymous substitution rates observed across *tat, vpu*, *env and nef *genes**. (a) dS across the *tat *gene. 'ess2'; exonic splicing silencer ESS2, 'ssa4b'; 3' splice acceptor site A4b. (b) dS across the *vpu *gene. 'n2', 'n3' and 'n4'; novel conserved sites. (c) dS across the *env *gene. "n1" is the novel conserved site. The black dotted horizontal lines indicate poorly aligned regions that were excluded from the analysis. "rre"; rev-responsive element, *; the 9 nucleotides (5' GACGGUACA 3') which bind to the Rev protein with highest affinity, "ss"; splice site region for the *tat *and *rev *3' exons. (d) dS across the *nef *gene. "G-A"; G-to-A hypermutations (see Additional file 1), 'var'; highly variable region, 'ppt'; poly-purine tract, "c"; PPT integrase attachment site, 'nre'; start of the negative repressive sequence, 'ets'; Ets-1 transcription factor binding site.

**Figure 5:**
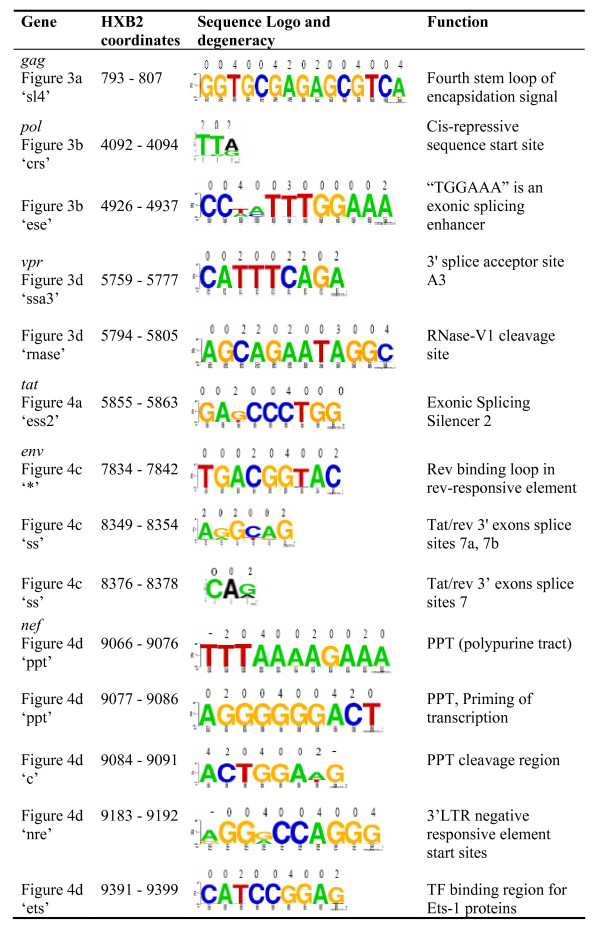
**Sequence motifs for the highly conserved regions with known function**. The fourteen regions with known specific functions found to be under strong purifying selection in HIV-1 genes. The range of coordinates on the HIV-1 genome for each motif is given in column 2. Numbers above each logo represent the degeneracy at each nucleotide site.

**Figure 6:**
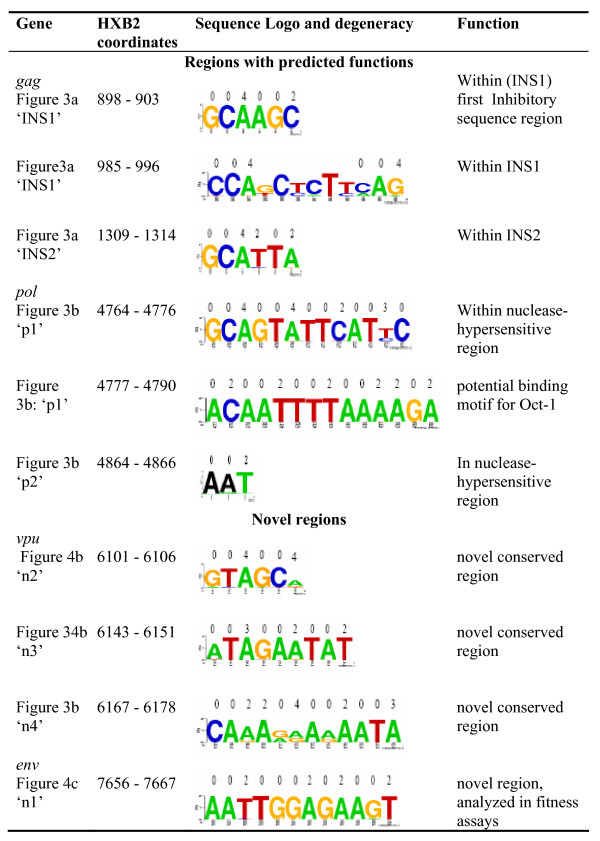
**Sequence motifs for the highly conserved regions with unknown specific function**. The five regions with predicted functions and four regions with unknown functions found to be under strong purifying selection in HIV-1 genes. The range of coordinates on the HIV-1 genome for each motif is given in column 2. Numbers above each logo represent the degeneracy at each nucleotide site.

We found evidence of strong purifying selection acting directly on the nucleotide sequence at twenty-three sites across the HIV-1 genome (Figures 3, 4, 5 and 6). Fourteen of these regions (marked in black in Figures 3 and 4) coincided exactly with well characterized functional motifs while for another five (marked in green in Figure 3), we were able to identify possible functions based on the known functions of the sequence domains in which they were situated. We could not, however, find plausible explanations for high degrees of sequence conservation observed within a twelve-nucleotide region of the *env *gene and three other regions in *vpu *(marked in red in Figure 4). Sequence logos illustrating the conservation in each of these twenty-three significantly conserved regions are shown in Figure 5 (for those with known specific function) and Figure 6 (for those with predicted and unknown functions).

The fourteen regions with known functions included one region consisting of fifteen nucleotides following the *gag *start codon, positions 793–807 of HXB2. This forms the fourth stem loop (sl4, Figure 3a) of the dimerization/encapsidation signal. The encapsidation signal is a four stem-loop structure which stretches from the 5' LTR and interacts with the nucleo-capsid protein, promoting formation of genomic RNA and blocking the initiation of transcription [14,36,37].

In the *pol *gene, we found two highly conserved regions with known functions. One corresponded to the first three nucleotides (HXB2 coordinate positions 4092–4094) of the 260 nucleotide long cis-repressive sequence (CRS; Figure 3b). The cis-repressive sequence inhibits expression of structural protein mRNAs by preventing their transportation from the nucleus – a process that is reversed by the rev-responsive element (RRE) [24-26]. The other was found at the 3' end of the intragenic nuclease hypersensitive domain (hs7 in Figure 3b). The latter motif, located between positions 4926 and 4937 and labeled "ese" in Figure 3b, is also located within HIV-1 exon 2 and the last six nucleotides, TGGAAA, of this conserved region form a known exonic splicing enhancer (ESE) of HIV mRNAs [38-42].

Two regions in *vpr*, a 3' splice acceptor site [41] and an RNase-V1 cleavage site [43,44] were also conserved (labeled ssa3 and rnase respectively in Figure 3d) at positions 5759–5777 and 5794–5805 respectively. An additional two highly conserved regions were observed within the *tat *gene, one containing an exonic splicing silencer, ESS2 between nucleotide positions 5855 and 5863 and the other at the 3' splice acceptor site A4b located at positions 5957 to 5968 (ess2 and ssa4b respectively in Figure 4a) [41].

Three of the highly conserved regions with known functions were in the *env *gene and included a nine-nucleotide long motif from position 7834 to 7842 within the RRE. The approximately two hundred nucleotides long RRE element within *env*, is known to interact with the Rev protein and facilitates the transport of late un-spliced and partially-spliced RNAs from the nucleus to the cytoplasm [19-21]. Although the RRE is associated with a long stretch of sequence that forms a well characterized secondary structure with various conserved domains [22], only the nine nucleotides that bind Rev with highest affinity [21,45] were sufficiently conserved to be detected using our conservative threshold (Figure 4c). Also conserved within the *env *gene were the two splice site regions at the end of the *tat*/*rev *exon, positions 8349–8354 and 8376–8378, usually referred to as 7a/7b, and 7 ("ss" in Figure 4c; [39,41,46,47]

The last four of the significantly conserved regions with known function were in the *nef *gene and coincided with the poly-purine tract (PPT), integrase attachment site, negative regulatory element (NRE) and Ets-1 transcription factor binding site (Figure 4d) (with HXB2 coordinates 9066–9083, 9084–9091, 9183–9192 and 9391–9399 respectively). The PPT precedes the start of the LTR and is known to associate with the 3' LTR, serving as a primer for the initiation of HIV-1 plus strand DNA replication [12,48-50]. A previous detailed RT RNase-H binding analysis revealed that priming of the plus strand occurs specifically at the 3' end of the PPT, at the "GGGGGG" motif [51-53]. The region adjacent to the 3' end of the PPT was also highly conserved. This region corresponds to the start of the 3'LTR and contains the cleavage site of the PPT by RNAse H as well as the start of integrase attachment region for the integration of the viral genome into the genome of the host [48,53-55]. Two codons in the central region of *nef *were highly variable, one dominated by G-to-A mutations in a sequence context consistent with APOBEC-induced hypermutations when compared to the Group M ancestral sequence [56] (see Additional file 1) and the second had a high rate of synonymous and nonsynonymous substitutions (labeled "G-A" and "var" in Figure 4d respectively).

One of the novel regions, the twelve nucleotide long motif in *env*, upstream of the RRE showed the highest degree of conservation of any region in the *env *gene ('n1' in Figure 4c; positions 7656–7667 of HXB2). Introduction of synonymous point mutations at positions 7, 9 and 12 in this nucleotide motif had no effect on virus output from transfected 293T cells (see Additional file 2(a)). No significant differences were observed between the wild type and mutated viruses with respect to their infectivity in M7-Luc cells (see Additional file 2(b)). Although the effect of mutations within this region is therefore not clear at present, future work making use of more sensitive competitive replication assays will determine whether these changes have an impact on viral fitness. Functional analyses of the three novel conserved regions in *vpu*, with HXB2 coordinates 6101–6106, 6143–6151 and 6167–6178 (n2, n3, n4 in Figure 4b) are being considered. The protein products of *vpu *and *env *are known to be produced from a bicistronic transcript [46] and the conserved regions in *vpu *may be involved in the control of translation.

In order to assess whether purifying selection is likely to cause false inference of positive selection we used standard methods to detect positive selection in a subtype C *gag *coding sequence alignment described previously [33]. Sites with ω significantly greater than one, implying positive selection, overlapped significantly with sites that had lower than average dS values (Fisher's exact test odds ratio = 4.4; p-value = 0.006; Additional file 3). This is consistent with a substantial proportion of the positive selection signals resulting from conservation of the synonymous sites rather than diversifying selection acting on the nonsynonymous sites.

## Discussion

This is the first study to provide a detailed analysis of site-to-site variation in the rate of synonymous substitutions across the HIV-1 genome. In the past, site-to-site variation in dS in HIV-1 has been investigated in a single gene [5,10] and in another study a single overall synonymous substitution rate for the entire genome was determined for comparison to other viral lineages [57]. We modeled site-to-site synonymous rate variation using a similar approach to a previous study [10], in that case only one HIV-1 gene, *vif*, was considered and sites that encode proteins in multiple reading frames were included. As a consequence, it was not clear whether the observed site-to-site rate variation resulted from variation in the synonymous rate or from selection acting on nonsynonymous substitutions in another reading frame. Here we focused primarily on regions of the HIV genome that encode proteins in a single reading frame and explored functions of nucleotide sequences that have the largest influence on synonymous substitution rate variation.

Previous studies have demonstrated that recombination causes false inference of positive selection. Since recombination affects tree topologies used in fitting phylogenetic models, it is also likely to cause biased estimates of dS. The recent development of methods to account for recombination in selection analyses [32] permitted us to remove recombination as a source of bias in our estimates of synonymous substitution rates.

In addition to the fourteen conserved regions with known functions and the novel sites in *env *and *vpu*, five conserved sites without previously reported specific functions occurred within known functional domains. These include three short (3–6 bp) motifs in the inhibitory (INS) sequence regions of gag (HXB2 positions 898–903, 985–996 and 1309–1314, Figure 3a). Previous *in-vitro *analyses have shown that short motifs within the approximately two hundred bp long INS regions, are responsible for the actual inhibition of mRNA expression [23,24,26]. Although the three motifs we find within this region have not been specifically identified *in-vitro*, a computational study by Wolff et al (2003) showed that the INS sequences have several short functional motifs within them [26]. The conserved sites we identified within INS1 and INS2 could serve the same inhibitory function. Functional assays elucidating the role of these sites in the inhibition of mRNA expression could help to determine the precise mechanisms by which inhibition occurs and whether these sites also play a role. In another previous study which analyzed the RNA secondary structure of the 5' region of HIV-1, these two regions, labeled 'INS1' in Figure 3a, were found to be involved in conserved Watson-Crick base-pairing [58]

The last two of the five conserved regions with unidentified specific functions were within the *pol *HS7. HS7 spans five hundred nucleotides, between positions 4481 and 4982 of the HIV-1 genome, and has an LTR-like regulatory function [16,17]. Previous studies revealed four domains towards the 3' end of this region (PU box, GC-box, site-C and site-D) that bind to specific transcription factors (TFs) and are also important for viral infectivity [16,17]. In these studies, the Oct-1, Oct-2, PU.1, Sp1 and Sp3 transcription factors were found to bind to at least one of the four identified sites. The two conserved regions we identified are outside these specific identified functional domains, but one of them at positions 4767–4790 (labeled "p1" in Figure 3b) showed potential binding to Oct-1 using the MATCH tool from the TRANSFAC database [59,60]. Potential association with a transcription factor was not observed for the three nucleotides (positions 4864–4866) labeled 'p2' and its adjacent sites.

Knowledge of regions of the genome that function at the nucleotide level is important for positive selection analysis. Conserved synonymous sites can cause false detection of positive selection and need to be either excluded from analyses or modeled appropriately. The danger is that some sites may be assumed to be evolving adaptively simply as a result of the purifying selection acting directly on the nucleotide sequence. We found evidence of this in subtype C *gag *sequences where positively selected sites significantly coincided with significantly low dS (Additional file 3). In addition, a study by Hurst & Pal (2001) also showed false detection of positive selection caused by purifying selection pressure acting on synonymous sites.

In many selection, studies the synonymous rate is assumed to be constant. However, negative selection acting on synonymous sites can potentially reduce gene-wide estimates of the synonymous rates below the neutral evolution rate. Comparison of site-specific nonsynonymous substitution rates against this underestimate of the neutral rate is likely to cause a proportion of the selectively neutral nonsynonymous sites to seem as though they are evolving adaptively. We have, however, used a very stringent cutoff to identify the twenty three regions within the HIV genome that have obviously reduced synonymous substitution rates. For a more conservative analysis of selection, all the significantly conserved sites, i.e., including those conserved at 95% confidence (p-value < 0.05, listed in Table 3) should be excluded from analyses along with sites that encode proteins in multiple frames. Surprisingly, we found that the rate of synonymous substitution, was higher, on average, in overlapping gene regions that encode proteins in more than one frame (p = 6 × 10^-7 ^from Wilcoxon rank sum test; Figure 1); however, lower dS was observed within some genome regions that are translated in multiple reading frames. Analysis of the most diverse sequences within subtype B and C revealed more highly conserved sites across the RRE and INS1 regions at the subtype level (Additional file 4). These putatively functional domains could also be removed in more conservative studies of selection acting on HIV protein sequences.

## Conclusion

We have analyzed sequence variation at the synonymous sites of non-overlapping regions of HIV-1 genes. We found substantial site-to-site variation in the rate of synonymous substitution with evidence of purifying selection pressure within functional domains such as the Rev-responsive element. The majority of conserved sites we identified are within functional regions that are well documented in the literature; however, the total number of sites that function at the nucleotide level is unknown and it is therefore difficult to assess the fraction of the known and novel functional sites that are detectable using our method. In addition to identifying putatively functional sites under purifying selection, these results contribute to the robustness of analyses of positive selection by identifying conserved synonymous sites that can cause false positive inference of selection. The sites presented in Table 3 and Figures 5 and 6 thus form a resource for future studies of selection pressures acting on HIV-1 genes.

## Competing interests

The authors declare that they have no competing interests.

## Authors' contributions

NKN carried out the analysis, interpreted the results and drafted the manuscript. KS provided HyPhy scripts, contributed to the methodology and participated in drafting the manuscript. PM carried out the biochemical fitness assays and participated in writing the manuscript. ZW and DM participated in validating the results and editing the manuscript. CS conceived and supervised the study and participated in writing the manuscript.

## Supplementary Material

Additional file 1 G-A mutations in a variable region in the *nef *gene. Mutations observed in reference sequences in comparison to Group M ancestral sequence identified using the hypermut tool available in the Los Alamos database. The highly variable region (labeled "G-A" in Figure 3d) showed G-A mutations and is boxed in red. Click here for file

Additional file 2 Functional analysis of a novel region in the *env *gene. (a) Production of p24 from transfected wildtype p81 and 3 mutants produced from synonymous mutations introduced in the previously uncharacterized conserved region in *env*. (b) Comparison of infectivity between wildtype and the mutants. Click here for file

Additional file 3 Evidence of overlap between high omega at a codon and low dS at the synonymous sites. Positively selected sites at which a significantly low dS was observed at the synonymous sites. Positively selected sites are shown in blue vertical lines and sites with low dS are shaded in light blue. Click here for file

Additional file 4 Highly conserved regions observed at the subtype-level. dS across subtypes B and C *gag *and *env *genes showing more conserved sites at the subtype sequence level within the INS regions in *gag *and RRE in *env*. Click here for file
