# Supplementary figures and images for: Extensive purifying selection acting on synonymous sites in HIV-1 Group M sequences
Source: Virol J. 2008 Dec 23;5:160. doi: 10.1186/1743-422X-5-160 (PMC2666660; doi:10.1186/1743-422X-5-160)

Additional file 1

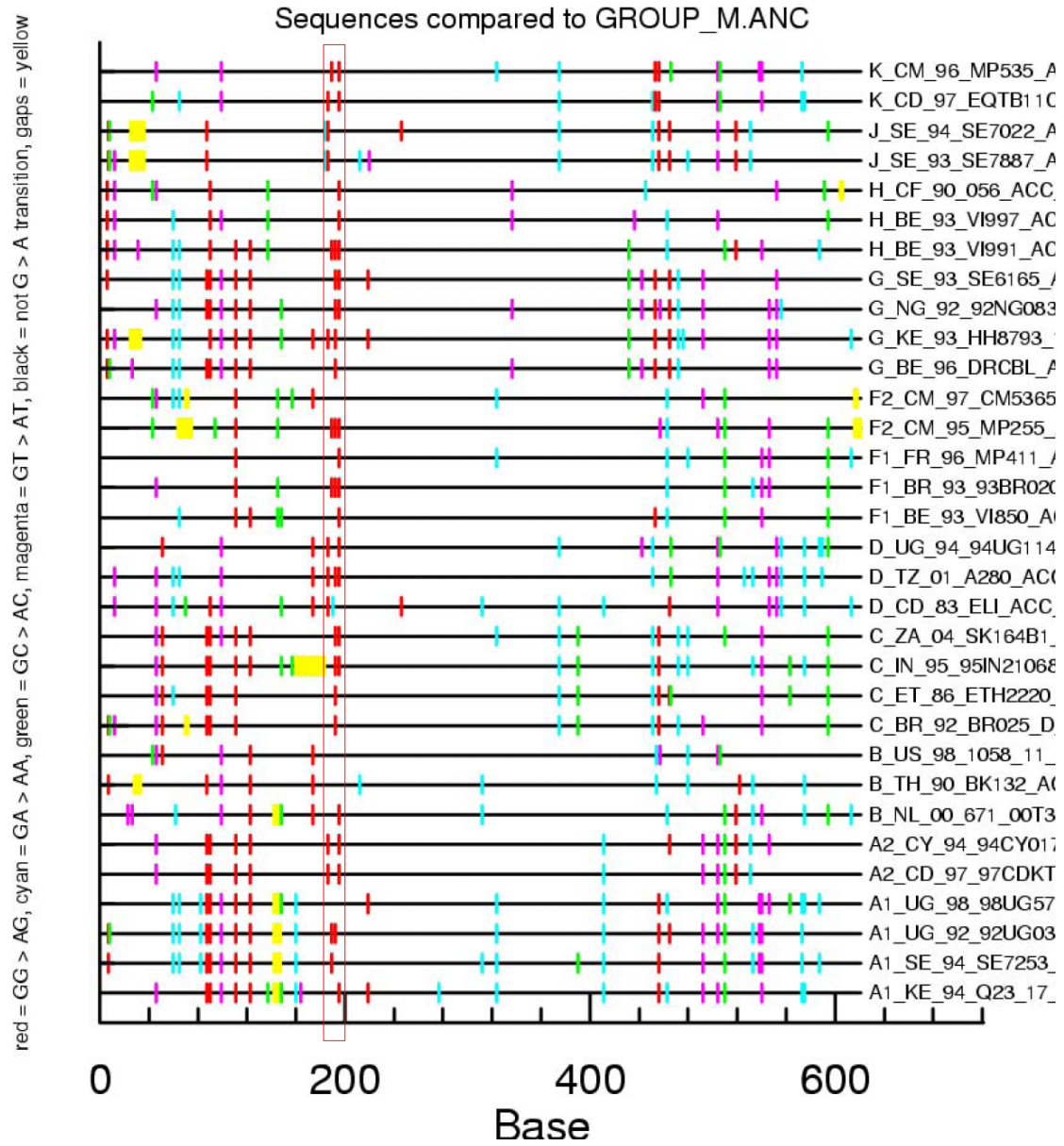

Supplement: Additional file 1 — G-A mutations in a variable region in the nef gene. Mutations observed in reference sequences in comparison to Group M ancestral sequence identified using the hypermut tool available in the Los Alamos database. The highly variable region (labeled "G-A" in Figure 3d) showed G-A mutations and is boxed in red. [file 1743-422X-5-160-S1.pdf]

Additional file 2

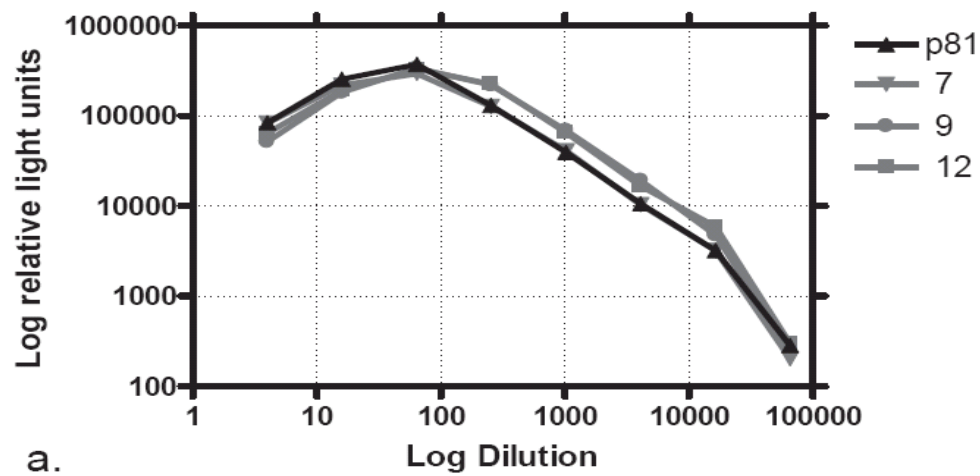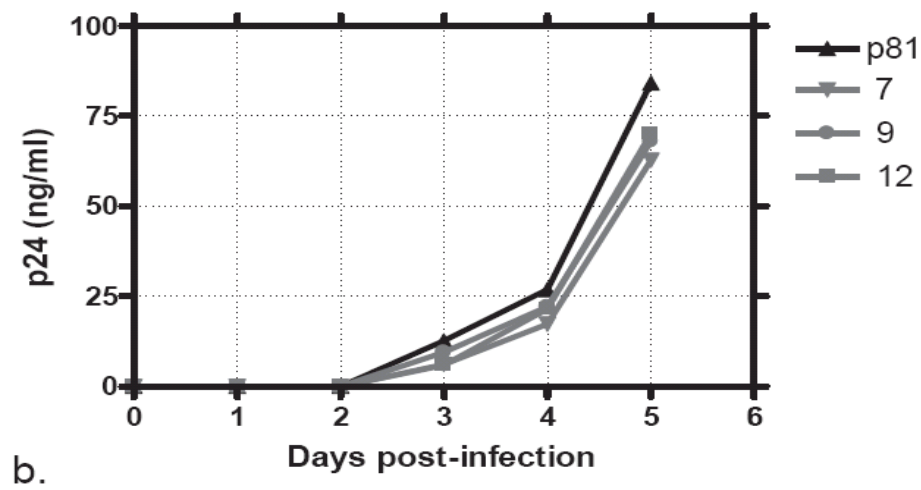

Supplement: Additional file 2 — Functional analysis of a novel region in the env gene. (a) Production of p24 from transfected wildtype p81 and 3 mutants produced from synonymous mutations introduced in the previously uncharacterized conserved region in env. (b) Comparison of infectivity between wildtype and the mutants. [file 1743-422X-5-160-S2.pdf]

Additional file 3

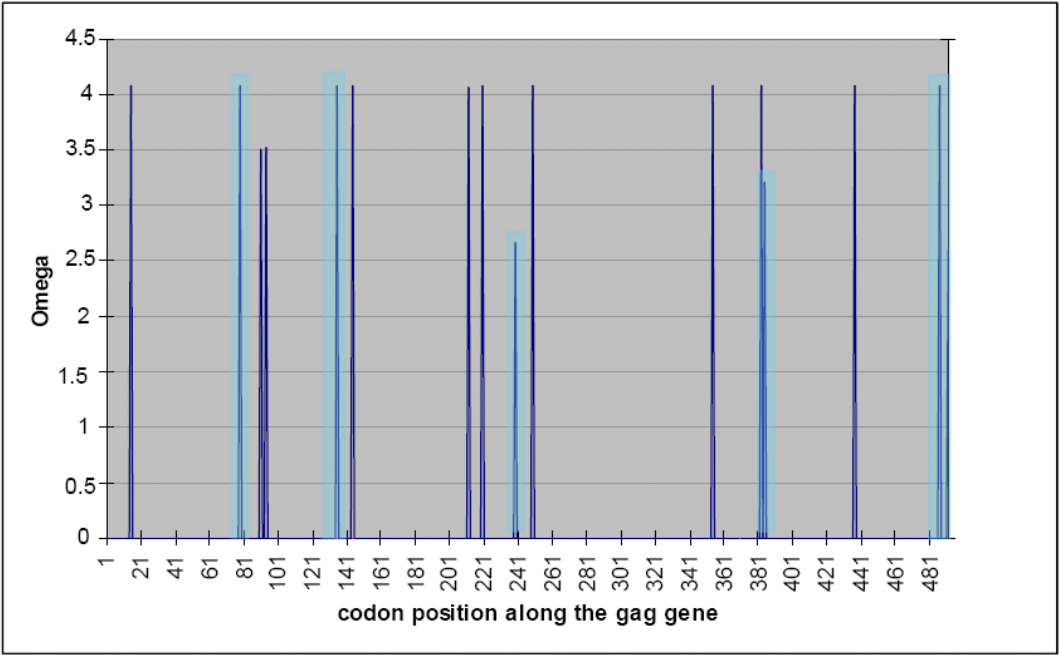

Supplement: Additional file 3 — Evidence of overlap between high omega at a codon and low dS at the synonymous sites. Positively selected sites at which a significantly low dS was observed at the synonymous sites. Positively selected sites are shown in blue vertical lines and sites with low dS are shaded in light blue. [file 1743-422X-5-160-S3.pdf]

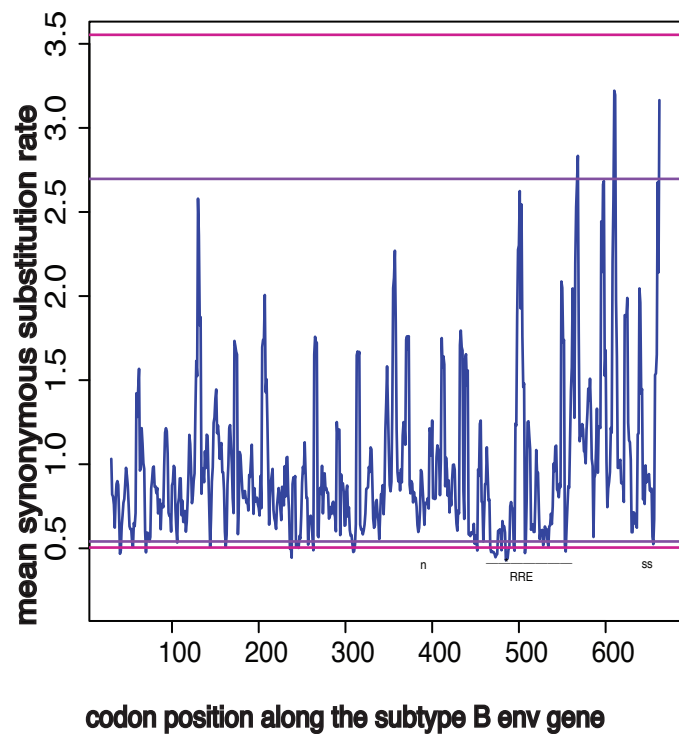

(a)

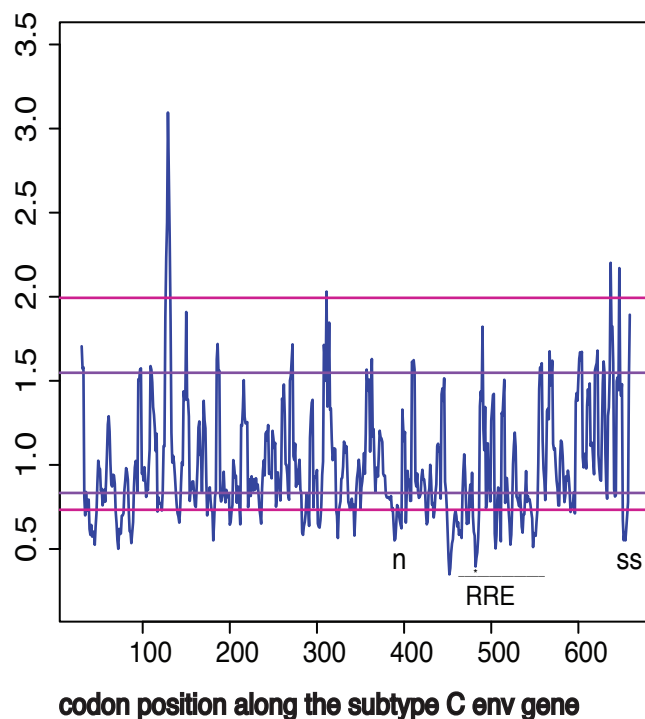

(b)

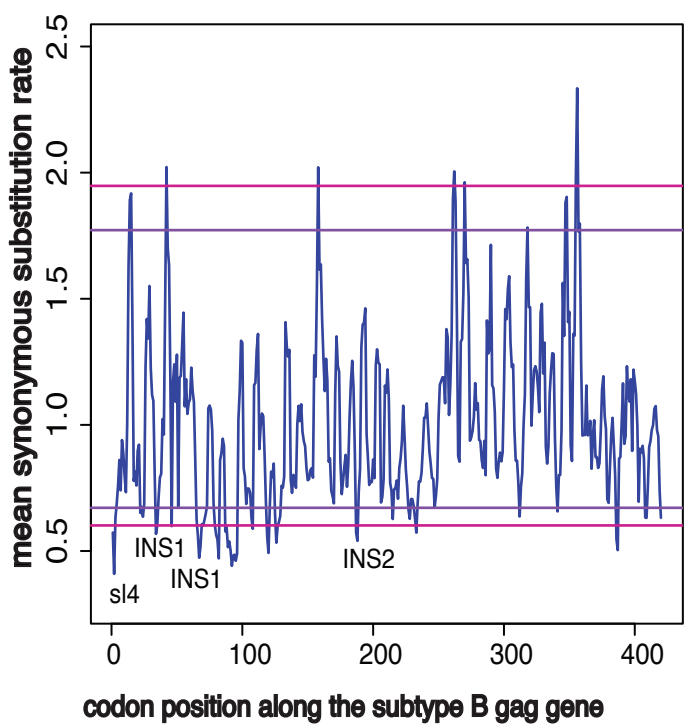

(c)

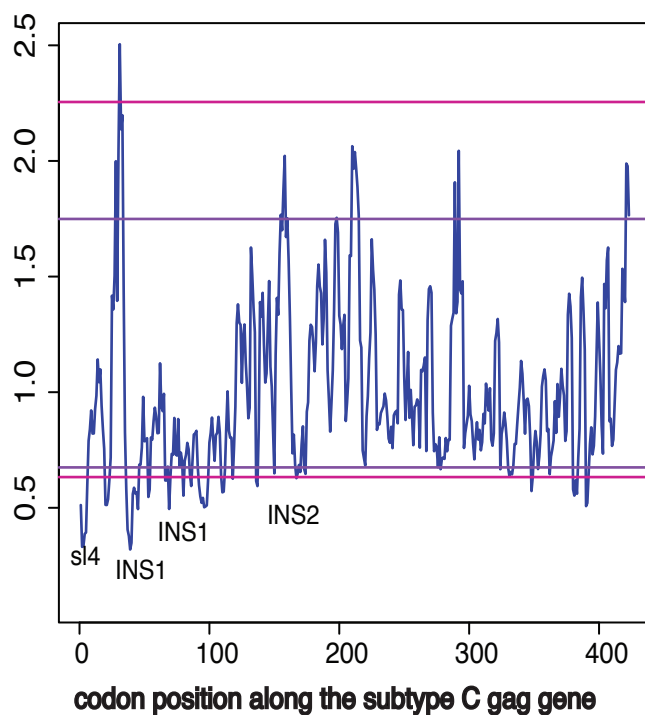

(d)

Supplement: Additional file 4 — Highly conserved regions observed at the subtype-level. dS across subtypes B and C gag and env genes showing more conserved sites at the subtype sequence level within the INS regions in gag and RRE in env. [file 1743-422X-5-160-S4.pdf]
